# P-358. Infection Prevention and Control Practice in Tertiary Care Military Hospitals in Bangladesh during COVID-19 Pandemic: Results from WHO IPCAF Assessment Tools

**DOI:** 10.1093/ofid/ofae631.559

**Published:** 2025-01-29

**Authors:** Syed Abul Hassan Md Abdullah, Md Golam Dostogir Harun, Md Shariful Amin Sumon, Ishrat Jahan, Md Mahabub Ul Anwar, Taufiq H Siddiquee

**Affiliations:** Bangladesh University of Professionals, Dhaka, Dhaka, Bangladesh; icddrb, Dhaka, Dhaka, Bangladesh; icddr,b, Dhaka, Dhaka, Bangladesh; Army HQ, Bangladesh Army, Dhaka, Dhaka, Bangladesh; Office of Health Affairs, West Virginia University, USA, Dhaka, Dhaka, Bangladesh; BRAC Healthcare, Dhaka, Dhaka, Bangladesh

## Abstract

**Background:**

WHO estimates a high burden of the MDR organism in hospital settings in South Asian countries^,^ especially in Bangladesh. Infection prevention and control (IPC) in healthcare settings is critical to limit the healthcare-associated infections, especially during pandemics like COVID-19. Military hospitals in Bangladesh are known for better compliance of health system practice. To measure current IPC level, activities, resources, and gaps, WHO developed the IPC Assessment Framework (IPCAF) assessment tools. We aimed to assess the IPC practices level in Military hospitals of Bangladesh to reduce the gaps.

**Figure:**
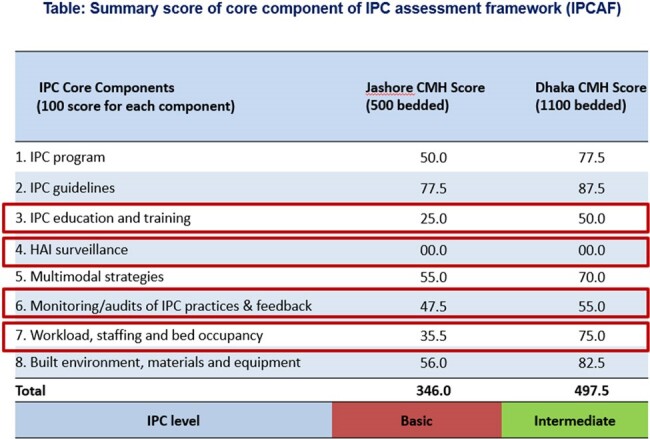
Table of WHO IPCAF score of Dhaka and Jashore CMH

**Methods:**

From Oct to Dec 2019, using IPCAF tools, we conducted a cross-sectional survey at two tertiary level Combined Military hospitals (CMH) in Bangladesh to collect the information. The hospital administrator, IPC focal person, or relevant person who had better knowledge about the overall IPC situation was interviewed. The tool comprises eight sections assessing the eight IPC core components. Based on the scores ranging from 0 to 100, the hospital was categorized into four distinct IPC levels - Advanced, Intermediate, Basic and Inadequate. Descriptive statistics were used to summarize and describe the key findings.

**Results:**

The mean IPCAF score was 421.75 out of 800.The Dhaka CMH (1100 bedded) was categorized as ‘intermediate’ with the score of 497.5 and Jashore CMH (500 bedded) scored 346 which was characterized ‘basic’ level. Both the hospitals scored very high in adherence of IPC guideline 87.5 and 77.5 respectively. Scoring of Dhaka CMH in IPC program (77.5), Workload, staffing & bed occupancy (75) and Built environment, materials and equipment (82.5) is higher than the same components of Jashore CMH. IPC education & training score of both hospitals (50 and 25 respectively) was not satisfactory. None of the hospitals had an IPC surveillance system with standard surveillance case definitions to track HAIs.

**Conclusion:**

Military hospitals needs to attain advanced level IPC practice. To achieve that organized quality improvement programs and feedback mechanisms is required. To strengthen all IPC core components, particularly IPC surveillance, monitoring, education & training is needed to improve for healthcare safety and resilience.

**Disclosures:**

**All Authors**: No reported disclosures

